# UV-induced translation capacity in *Fusarium mangiferae*

**DOI:** 10.3389/ffunb.2026.1965588

**Published:** 2026-10-26

**Authors:** Quyen Hoang, Shira Milo, Dibya Mukherjee, Shay Covo

**Affiliations:** Department of Plant Pathology and Microbiology, Institute of Environmental Sciences, Robert H. Smith Faculty of Agriculture, Food and Environment, The Hebrew University of Jerusalem, Rehovot, Israel

**Keywords:** DNA damage response, UV damage repair, translation, ribosome biogenesis, *Fusarium*

## Abstract

**Introduction:**

In fast-growing cells, most RNA molecules encode ribosomal subunits. During growth arrest caused by DNA damage, ribosome biogenesis decreases because it is highly energy-demanding. This was observed in cells from bacteria, yeast, and humans.

**Results:**

Here, we report that in *Fusarium mangiferae*, UV radiation significantly induces ribosome levels and translation capacity. This UV-induced response occurs after filament formation. Translation induction modestly but significantly enhances both UV survival and repair. While some mRNAs differentially associated with ribosomes after UV exposure are linked to DNA repair, most belong to modules involved in protein folding and gene expression. TOR signaling is required for the immediate UV induction of ribosome biogenesis genes. However, we provide evidence that UV induces mechanisms that operate upstream of, or in parallel with, TOR.

**Discussion:**

These findings suggest a novel type of response to DNA damage, in which the primary aim is to protect gene expression from the detrimental effects of UV radiation on transcription.

## Introduction

Ultraviolet (UV) light is an important determinant in the ecology and physiology of both prokaryotic and eukaryotic microbes (Albarracín et al., 2012; Palmer et al., 2018). While UV can damage several biochemical components, probably the most sensitive is DNA, since mutants in DNA repair are extremely sensitive to UV (Cleaver, 2016). The most common UV lesions are cyclobutane pyrimidine dimers (CPDs) and 6–4 UV photoproducts, both of which significantly distort the DNA helix (Rastogi et al., 2010). This severe disruption of DNA structure interrupts DNA replication, leading to stalled DNA replication forks and cell death. UV is also mutagenic; primarily, it causes point mutations, most commonly C-to-T transitions (Laughery et al., 2020). In addition to its negative effect on DNA replication, UV inhibits transcription (Nieto Moreno et al., 2023).

Several DNA repair mechanisms suppress the genotoxic and mutagenic effects of UV. These mechanisms can be divided into those that remove lesions from the entire genome and those that remove lesions from transcribed genes. UV damage repair mechanisms in ascomycete fungi include UV damage endonuclease (UVDE) and photolyase, which both specifically recognize UV lesions and act globally across the entire genome (McCready et al., 2000; Goldman et al., 2002; Inoue, 2011). Nucleotide excision repair (NER) recognizes DNA helix distortions by scanning the genome using the proteins Ddb1 and Xpc (global NER) (Lindahl et al., 1997; Moser et al., 2005; Scrima et al., 2011). Alternatively, the lesions are recognized by stalled RNA polymerase II with the assistance of proteins Csa and Csb, a process known as transcription-coupled (TCR) NER (Lindahl et al., 1997; Lindahl and Wood, 1999; Sancar and Reardon, 2004; Moser et al., 2005). TCR facilitates the resumption of transcription following UV exposure more rapidly than if only global DNA repair mechanisms were operating. UV-induced transcription stalling is probably a severe challenge, since eukaryotic cells evolved a last-resort solution to this problem by degrading stalled RNA polymerase II. In fact, TCR is also required for the release of stalled RNA polymerase I (Daniel et al., 2018; Nieto Moreno et al., 2023).

The bacterial SOS response is the model of the cellular response to UV (Maslowska et al., 2019). During SOS activation, there is an orchestrated induction of UV repair and mutagenesis genes. The SOS response induces, at the mRNA level, the expression of DNA repair, recombination, and DNA damage tolerance genes, some of which increase up to 100-fold (Maslowska et al., 2019). A comprehensive, SOS-like induction of DNA repair genes has never been demonstrated in fungi. Nevertheless, some DNA repair/DNA damage tolerance genes are induced by UV in *Saccharomyces cerevisiae*, *Schizosaccharomyces pombe*, and *Neurospora crassa* (Davey et al., 1997; Sakuraba et al., 2000; Fu et al., 2008). Several attempts were made to characterize the UV response in various eukaryotic organisms under different exposure and recovery conditions. In general, the transcriptional response was variable and complex, highlighting responses to reactive oxygen species, flavonoid biosynthesis, and cell cycle control (Wade et al., 2009; Pontin et al., 2010; Guo et al., 2019; Milo-Cochavi et al., 2019a; Milo et al., 2024). A major part of the eukaryotic response to UV and other forms of DNA damage is cell cycle arrest, which allows cell division to occur only when genome integrity is not severely compromised (Carr, 2002; Sertic et al., 2012). Thus, segregation of partly replicated DNA into the daughter cells is avoided.

In many aspects, the cellular response to DNA damage is a particular case of stress response that leads to growth arrest. In fast-growing yeast cells, up to 60% of genes are involved in ribosome biogenesis; however, this process is resource-intensive. Therefore, underexpression of ribosomal proteins is a sign of growth arrest (Warner, 1999). Translation is reduced in response to UV and oxidative stress, which also causes DNA damage (Ying and Khaperskyy, 2020; Picazo and Molin, 2021; Meydan et al., 2023). The effect of DNA damage on ribosome biogenesis was demonstrated at the cellular and molecular levels. In yeast, Xbp1, which suppresses the expression of ribosomal proteins and ribosome biogenesis, is activated by the DNA damage response (Tkach et al., 2012; Miles et al., 2013). In addition, UV-induced DNA lesions specifically cause dissociation of yeast RNA polymerases I (Tremblay et al., 2014). Further evidence supports a link between DNA repair and ribosome biosynthesis (Ogawa and Baserga, 2017). In humans, under normal growth conditions, several DNA repair proteins, such as the Ape1 endonuclease and the Werner syndrome helicase, are localized to the nucleolus. They are thought to be involved in ribosome biogenesis. Once the DNA damage response is triggered, these repair proteins dissociate from the nucleolus (Lirussi et al., 2012). The opposite is also true: some ribosome biogenesis genes that leave the nucleolus following DNA damage participate in DNA repair (Ogawa and Baserga, 2017).

Here we present our study on the UV response of the plant pathogen *Fusarium mangiferae* (Marasas et al., 2006; Michielse and Rep, 2009; Ploetz, 2015). The life cycle of this fungus starts with the germination of a single cell, leading to development from a single-nucleus spore (conidium) to a filamentous hypha. With time, the hypha elongates and branches to form a developed mycelium (Li et al., 2025). Only a few of the nuclei within the mycelium are mitotically active; the rest are dormant (Ruiz-Roldán et al., 2010). Based on these differences between fungal spores and filaments, we studied the response to UV at different stages of fungal development.

We previously reported the analysis of the transcriptomic response of *F. mangiferae* and *F. oxysporum* to UV under two developmental stages: 8 and 14 hours post-inoculation (hpi), while in the former stage, germination occurs with only one or two nuclei; in the latter stage, the filament is established with 10 or more nuclei (Milo-Cochavi et al., 2019a; Milo et al., 2024). As expected, the expression of genes related to translation was downregulated following UV irradiation of fungi at 8 hpi. Surprisingly, we observed a remarkable induction of translation-related genes in both fungi when they were irradiated at 14 hpi (Milo-Cochavi et al., 2019a; Milo et al., 2024).

There are two nutrient-sensing pathways that induce ribosome biogenesis: the target of rapamycin (TOR) and the cAMP-activated kinase PKA (Guerra et al., 2022; Plank, 2022). There is considerable crosstalk and redundancy between the pathways, as well as some specificity. Both pathways activate, among others, Sfp1 and Stb3, which drive ribosome biogenesis, and deactivate Tod6 and Dot6, which suppresses ribosome biogenesis (Plank, 2022).

Here, we show that ribosome abundance is markedly increased in response to UV and that translation capacity is markedly induced in both fungi. We found that UV-induced translation capacity is important for both UV repair and survival. Our research proposes a novel strategy for responding to UV lesions, about five decades after SOS was identified in bacteria.

## Materials and methods

### Fungal isolates and culture conditions

Spores of *F. mangiferae* (strain MRC7560, isolated in Israel) and were incubated in KNO3-based medium (KNO3: 10.1g, Sucrose: 30g, YNB (Yeast Nitrogen Base): 1.7g in 1L distilled water) on an orbital shaker (250 rpm) at 28 °C for 7 days. Microconidia were obtained by filtering the culture through a nylon cell strainer (40 µm mesh, Corning, USA) and resuspended in sterile water. Conidia were then inoculated into potato dextrose broth (PDB; BD Sparks, USA) at 1x10^8^ conidia/mL. The cultures were incubated on an orbital shaker at 28 °C for 8 or 14 h, then harvested immediately or treated as specified.

### UV irradiation

Spores or germlings of *F. mangiferae* (0, 8, and 14 h post-inoculation) were obtained by filtering 100 mL of the cultures. Suspensions containing 2 x 10^7 conidia/mL were irradiated with different UVC doses ranging from 30 to 200 J/m^2^ (254 nm, 15 W lamp, Osram, Germany); the incident fluence was measured with a radiometer (YK-35UV, Digital Instruments, USA). Irradiation dose and incubation time after irradiation were determined in downstream experiments (RNA sequencing, liquid holding, immunodot blot, ribosome profiling, or PUNCH-P). Samples were harvested, immediately flash-frozen in liquid N2, and kept at -80 °C until further experiments.

### Ribosome isolation

Frozen samples were ground into a fine powder using a mortar and pestle in liquid nitrogen. Cell lysates were prepared in a cold lysis buffer containing 50 mM Tris-HCl (pH 7.5), 50 mM KCl, 10 mM MgCl_2_, 1% protease inhibitor cocktail (Roche) supplied with cycloheximide (10µg/mL, final concentration) homogenized with glass beads using a Mini bead beater, followed by two cold centrifugations: the first one at 5000 rpm, 4 °C for 5 minutes and the second one at 12000 rpm for 30 minutes. The lysate supernatant was then transferred into ultracentrifuge tubes, and 5 mL of 32% sucrose (in lysis buffer) was carefully added using the long-range needle. Equal amounts of nucleic acids (25 A254i units) were loaded. Samples were spun in a Beckman Colter 70.1 Ti rotor at 50.000 rpm for 16 hours at 4 °C. After discarding the supernatant, the resulting ribosomes, forming a clear pellet, were resuspended in 200 μL extraction buffer (ribosome recovery) (Mehta et al., 2012). The pellet is enriched for ribosomes (**Supplementary Figure S1**).

### Ribosome fractionation and RNA extraction

Ribosome fractionation was carried out following the ribosomal isolation (dissolved pellet, above). 200 μL of the dissolved pellet was loaded onto a 12 mL linear sucrose gradient (10-50%) prepared in lysis buffer. The sucrose gradient was prepared using a gradient maker (Gradient Master™ from BioComp) according to the manufacturer’s instructions. The loaded samples were then centrifuged at 39,000 rpm for 3 h at 4 °C using an SW 41Ti rotor. Individual fractions were collected with a fraction collector. RNA was precipitated from 0.5 mL fractions by mixing each fraction with an equal volume of 6 M guanidine thiocyanate and 2 volumes of 100% ethanol, then incubating overnight at -20 °C. The RNA was pelleted, washed, and briefly resuspended: it was pelleted in 75% ethanol by centrifugation for 30 minutes at 10,000 g at 4 °C, and the supernatant was discarded. Then the pellet was washed using the same procedure with some differences. 0.5–1 mL of 75% ethanol was added to the pellet, which was then vortexed for a few seconds. Next, the pellets were incubated for 10–15 minutes at room temperature to dissolve residual guanidinium, then centrifuged for 5 minutes at 10,000 g at 4 °C, and the supernatant was discarded. Then the pellet was air-dried. Finally, the pellet was dissolved in 100 µL of either DEPC-treated water or freshly deionized formamide (For Formaldehyde gel electrophoresis). By incubating for 10–15 minutes at 60 °C to ensure complete solubilization. The samples were run by formaldehyde agarose gel electrophoresis (1.5% agarose, 5% formaldehyde in 1 × MOPS buffer (20 mM 3-[N-morpholino] propane-sulfonic acid (MOPS, M3183, Sigma), 5 mM sodium acetate, 1 mM EDTA)). 4.7 μL of RNA was mixed with 11.3 μL sample buffer (for 1.13 mL: formamide 660 μL, MOPS buffer 10X 200 μL, formaldehyde 37% 270 μL), then heated to 60 °C for 5 minutes, cooled on ice, and 4 μL of loading dye was added. Gels were run for 1.5–2 h in an ice bucket. The gels were stained with SYBR Gold (Invitrogen, S11494) by the standard procedure. Gel images were captured with a Gel Doc™ XR+ (Bio-Rad), which provides a 3-order-of-magnitude dynamic range. ImageJ software was used to determine band intensities and, consequently, the ribosome signal. The range of signal intensity from the faintest to the strongest in all gels was in one order of magnitude. For each gel image, the relative intensities of the different gradient fractions were determined. Next, the fractions with the highest intensities from each gel were run together on a single gel with appropriate dilutions. After image acquisition, the signal intensity was measured using ImageJ and assigned a relative intensity value compared with the unirradiated and untreated sample (signal intensity as 1). Finally, the relative intensities of all fractions across all gels were recalculated relative to a signal intensity of 1. In addition, the A254 nm absorbance of the RNA isolated from each fraction was determined using a quartz 96-well plate. To calculate the area under the curve, we used the Trapezoidal Rule Guide to approximate the segments between X and Y data points: =(Y3+Y2)/2*(X3-X2). Y are absorbance values, and X are fractions of the gradient.

For RNA-seq experiments, fractions from 3–12 and 13–24 were combined and processed for RNA extraction using a Trizol LS 3:1 ratio according to the manufacturer’s instructions. After RNA extraction, we followed a standard next-generation sequencing workflow.

### Transcriptome analysis

Raw reads were trimmed using Fastp 38 and assessed for quality using FastQC (https://www.bioinformatics.babraham.ac.uk/projects/fastqc/). Ribosomal RNA gene sequences of *Fusarium fujikuroi* (EF1) were downloaded from ENSEMBL Fungi Biomart (http://fungi.ensembl.org/index.html) and used to construct a reference database for FMN. Raw reads were mapped against the reference rRNA database using HISAT2 39. Mapped reads were discarded, whereas unmapped reads were collected and remapped against the *F. mangiferae* [FMN] MRC7560 [GCA_900044065.1] reference genome. Mapped reads were then counted with HTSeq-count (Chen, 2023). The reads were assembled and normalized, and gene expression levels were quantified using the DESeq2 R package (Zhang et al., 2021). Differential expression was assessed for genes expressed in all combinations of UVC treatments vs. untreated controls. DESeq2 was used to identify differentially expressed genes using the negative binomial distribution, with adjusted P-values (P < 0.05) and log2 fold changes> +1 or < -1. To identify significantly enriched genes in the ribosomal fraction only after UV exposure, ribosomal-enriched genes with and without UV were analyzed using a Venn diagram in Venny (https://bioinfogp.cnb.csic.es/tools/venny/).

### Functional enrichment and network analysis

Functional enrichment analysis was performed using the gprofiler2 v0.2.3 R package 40 and one-tailed Fisher’s exact test. The g: SCS method was used to compute multiple-testing correction, with P < 0.05 as the significance threshold (Kolberg et al., 2023a). To reveal the genes that are uniquely expressed under distinct conditions, either up- or downregulated differentially expressed genes from different ribosome fractions were intersected based on condition (treated vs. untreated). Differences in each set (unique in treated or unique in untreated) were analyzed using the protein-protein interaction network analysis in the STRING database (Kolberg et al., 2023b) and visualized in Cytoscape v3.9.1 (https://cytoscape.org/index.html). Clusters were identified and annotated using the Cytoscape AutoAnnotate plugin v1.3.5 (http://baderlab.org/Software/AutoAnnotate).

### PUNCH-P and western Blot

The PUNCH-P protocol was performed as described in (Aviner et al., 2013). 5 A260 units of Ribosomal proteins were used for each labeling with Biotin-Puromycin. 100 pmol of Biotin-dC Puromycin (Jena Biosciences cat# NU-925-BIO-L) was added per 1 OD260 unit of ribosome-containing solution. As a control, non-puromycylated (-P) reactions were also used. The reactions were incubated at 37 °C for 30 minutes, after which the proteins were precipitated with chloroform and methanol to remove excess Puromycin for subsequent SDS-PAGE. Briefly, after the reaction, water and 6x SDS buffer containing bromophenol blue were added to a final volume of 150 µl. Following the general protocol for protein precipitation with 4 volumes of methanol, 2 volumes of chloroform, and 3 volumes of water. The pellet will have some blue color from the Bromophenol blue for easy recognition. The protein pellet was then resuspended in SDS buffer and loaded onto a 12% SDS PAGE. Proteins were transferred onto an Amersham Protran 0.45 μm nitrocellulose membrane (GE Healthcare, 10600003) at 25V overnight using the wet transfer method. The membrane was stained with Ponceau S solution (2% solution in 2% acetic acid) to visualize transfer efficiency. The membrane was washed off this stain with water for 5 minutes, followed by a 5-minute wash with TBST (Tris-buffered saline with 1% Tween-20). The membrane was blocked with 5% BSA in TBST for 1 hour at room temperature. After blocking, the membrane was washed three times, each for 5 minutes at room temperature in TBST, to remove any remaining BSA solution. The membrane was then incubated with a 1:5000 dilution of streptavidin-horseradish peroxidase (HRP) (Sigma SA10001) in TBST for one hour at room temperature.

### Liquid holding assays

Conidia, germlings, or filaments were kept in distilled water added with Actinomycin D (Sigma, A9415) (500 nM or as described in the figures), Rapamycin (Thermofisher Scientific, J62473.MF), Rbin2 (Sigma, 2032282) (1 µM), Cycloheximide (Sigma, 01810) (0.3 µg/mL), and held for different incubation times before plating on PDA plates. Colonies were counted after 2 to 3 days post-inoculation, and the suitable concentration with the best holding time that showed 100% survival was chosen for further experiments. After UV irradiation, conidia or germlings were kept in distilled water added with desirable doses of Actinomycin D, Rapamycin, Rbin2, Cycloheximide, and held for the indicated time to check the effect of drugs on survival under UV. Colonies were counted after 2 to 3 days of inoculation, and the additive effect would be evaluated. At least three sets of experiments were repeated, each with three biological replicates. The same procedure was used also to test the effect of the different inhibitors on ribosome biogenesis and translation capacity (PUNCH-P protocol).

### Immunodot blot assay

After UV irradiation, samples were kept in distilled water containing Actinomycin D for the indicated time (6 to 24 h), then harvested and processed for DNA extraction using the CTAB-based method. In short, samples were ground into a fine powder using liquid nitrogen and disrupted with a Minilys bead beater for 60 s at medium speed in RLC buffer. Genomic DNA was purified using 2% (wt/vol) hexadecyltrimethylammonium bromide (CTAB) buffer, followed by a chloroform-isoamyl alcohol (24:1 [vol/vol]) phase separation procedure and precipitation in a final concentration of 50% ice-cold isopropanol. An immunodot blot assay was used to quantify DNA lesions, as described previously. Briefly, after denaturation at 95 °C for 10 minutes, 400 ng of DNA was combined with an equal volume of 2 M ammonium acetate and placed on ice. Each DNA sample was spotted onto a nitrocellulose membrane (soaked in 1 M ammonium acetate buffer for 10 minutes at room temperature), using a Bio-Rad dot blot manifold. The membrane was washed twice with 1 M ammonium acetate buffer and once in 6X saline sodium citrate buffer. Membranes were dried in a vacuum gel dryer (model 583; Bio-Rad, USA) for 90 minutes at 80 °C. After blocking in 5% powdered milk in PBST (1 mL Tween 20, 100 ml of 10× phosphate-buffered saline [Biological Industries, Israel], 899 mL double-distilled water), membranes were probed with mouse anti-CPD antibody (NMDND001; Cosmo-Bio, Japan). Following secondary antibody application (peroxidase-conjugated; Jackson Immuno Research Laboratories, USA), enhanced chemiluminescence was used to detect the antibody-dependent signal from each DNA spot on film. The intensity of each spot was quantified using ImageJ.

## Results

### Transcriptome analysis of *Fusarium mangiferae* irradiated with UV 14 hpi reveals induction of ribosome biogenesis genes

Using network analysis, we reanalyzed the RNA-seq data for *F. mangiferae* irradiated with UV at 14 hours post-inoculation (hpi), grouping 0, 30, 60 minutes recovery times (Milo et al., 2024). Among the biological modules that were most significantly induced by UV at 14 hpi were translation and ribosome biogenesis (**Supplementary Figure S2**). This observation is surprising, since exposure to DNA damage in *Fusarium* is usually associated with reduced ribosome biogenesis due to its negative effect on cell growth (Tremblay et al., 2014; Milo-Cochavi et al., 2019a; Milo-Cochavi et al., 2019b). Therefore, we first confirmed that UV inflicts growth delay under these conditions. Without irradiation, the number of nuclei in the filament increased steadily every two hours throughout the experiment. When filaments were irradiated at 14 hpi, *F. mangiferae* showed no significant change in the number of nuclei during the first 4 hours (**Supplementary Figure S3**). In addition to ribosome biogenesis genes, we found that pyrimidine biosynthesis and mitochondrion-related processes were both upregulated at 200 J/m^2^ exposure (**Supplementary Figure S2**). Modules related to protein degradation were downregulated when cells were exposed to 200 J/m^2^ (**Supplementary Figure S2**). The increase in expression of pyrimidine biosynthesis, mitochondrial-related, and ribosome biogenesis genes following UV exposure suggests that rNTPs are consumed for substantial ribosome assembly.

### UV induces ribosomes in *Fusarium mangiferae* filaments

To test whether irradiation causes ribosome buildup, we irradiated 14 hpi filaments and collected samples 2 hours after irradiation. If UV induces ribosome biogenesis, then the ribosomal fraction (excluding free mRNA or tRNA) should increase. To test this, we first enriched the ribosomal fraction from the extract by ultracentrifugation through a 35% sucrose cushion (See Materials and Methods, **Supplementary Figure S1**). Although equal amounts of RNA were loaded on the sucrose cushion, the pellet concentration was higher after 2 hr recovery from 200 J/m^2^ by 1.3-fold ± 0.05 SD (per experiment, the absolute RNA concentrations varied; however, the ratio between irradiated and unirradiated was stable). Next, the dissolved pellets were separated on a 10- 50% sucrose gradient; after fraction collection and RNA precipitation, the concentration of the RNA was determined in each fraction. As shown in **Figure 1A**, the absorbance peak of the irradiated samples was higher, wider, and towards the bottom than that of the unirradiated samples. These pattern differences were consistent across four independent experiments in which irradiated and non-irradiated cultures were processed side by side (although absolute absorbance varied between experiments). We therefore calculated the area under the curve for each experiment. The ratio between irradiated (200 J/m^2^) and non-irradiated was 1.28 ± 0.19 (1.19, 1.57, 1.20, 1.16). We note that the absorbance pattern we show differs from the classical 80S, disome, polysome pattern; this is due to our experimental setup and available equipment. Because we measured a low A254 absorbance at the bottom of the gradient, we determined whether we could detect ribosomal RNA bands across the gradient fractions. To do so, we ran an agarose gel as described under Materials and Methods. We measured the ribosomal RNA band intensities for each fraction (see Materials and Methods). We found that following irradiation, the amount of ribosomes in *F. mangiferae* increased (**Figure 1B**), even at a low UV dose (50 J/m^2^). Specifically, we observed increased RNA band intensity corresponding to ribosomes in the bottom fractions of the gradient, indicating an increase in polysomes. We observed this pattern across four independent experiments (two shown in **Figures 1B**, **2B**). We note that our experimental approach does not allow us to determine whether UV specifically increases the polysomal fraction.

**Figure 1:**
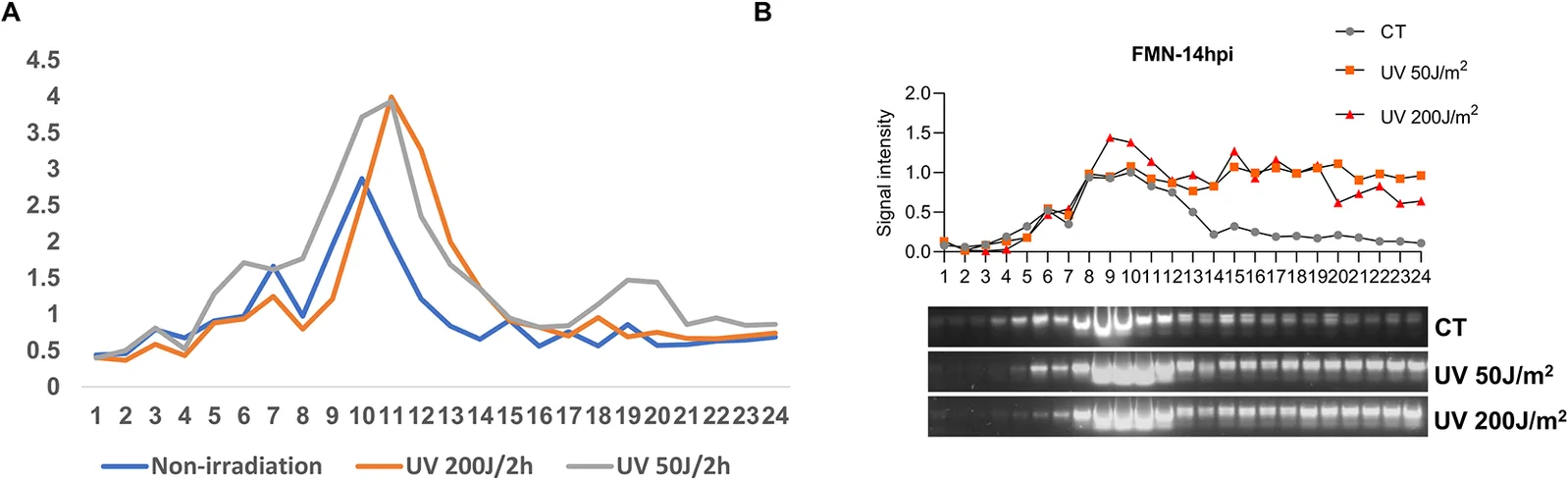
UV induces ribosome biogenesis in *F. mangiferae*. Conidia of *F. mangiferae* (FMN) were inoculated for 14 h (filaments) in PDB medium. Fungal filaments were irradiated with UV or not (CT). The fungi were recovered for two hours in water. After recovery, the cultures were lysed and loaded onto a sucrose cushion, then separated on a gradient as described under Methods. The RNA samples from each fraction were precipitated as described in Materials and Methods. **(A)** The A254 absorbance was determined (Y axis) for each fraction (X axis). **(B)** 4 μL from each fraction was loaded on the gel to 2% agarose gels. The images were captured by a GelDoc™ from Bio-Rad, which allows a three-order-of-magnitude dynamic range. Next, the relative ribosome intensity in the entire experiment was calculated based on the strongest band signal in the control sample (signal intensity as 1). This was done by comparing the signal of the fractions with the strongest ribosomal bands within each gel, on a separate gel (described in detail under Materials and Methods).

**Figure 2:**
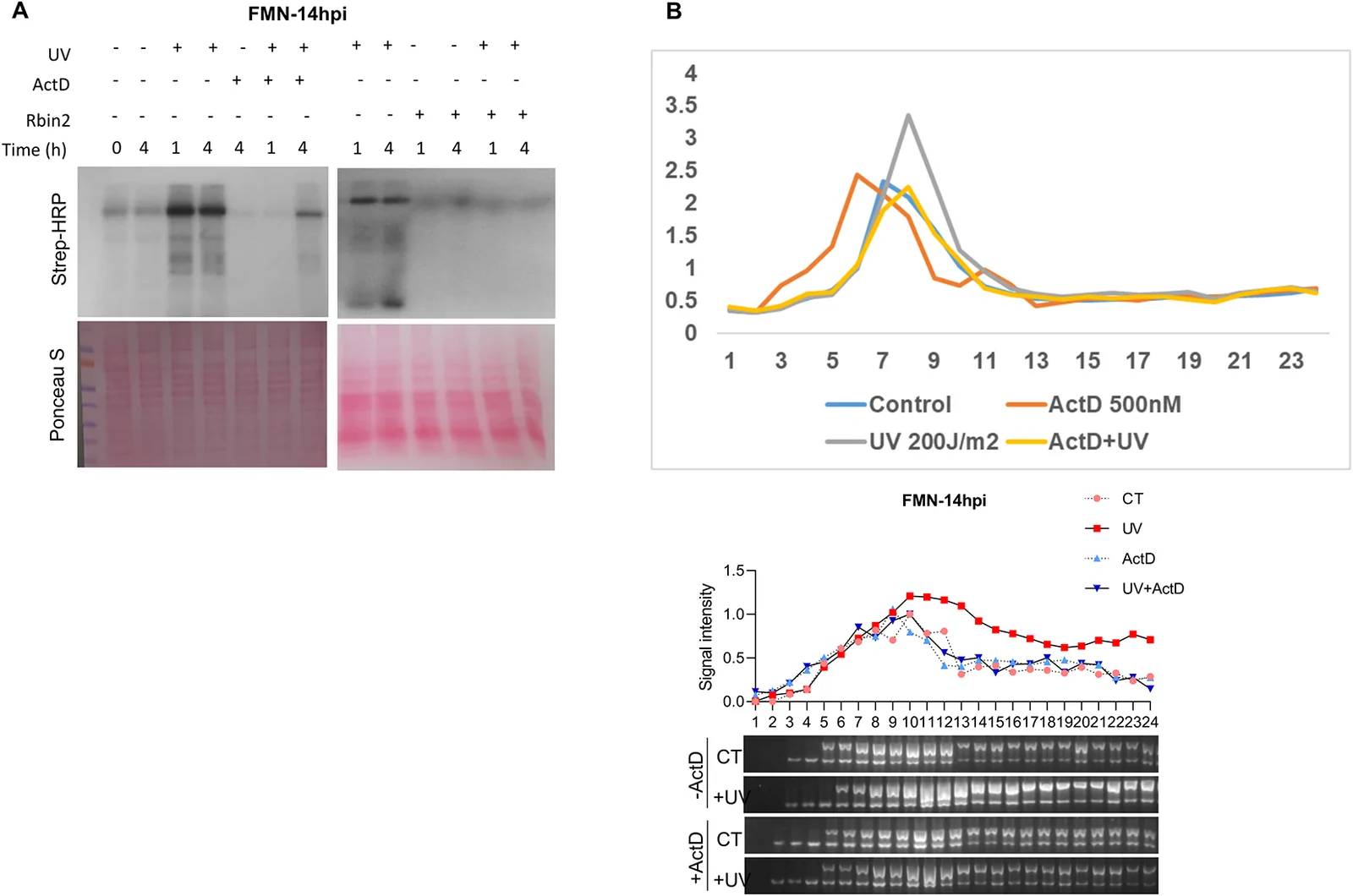
UV-induced translation capacity occurs at the ribosome transcription and maturation levels. 14 hpi *F. mangiferae* (FMN) filaments were irradiated or not with 200J/m^2^ UV. UV recovery occurred for 2 h in water under the conditions specified below. Ribosomes were separated from the extracts using a sucrose cushion (see Methods). **(A)** The purified ribosomes were incubated with Biotin-Puromycin to label nascent peptide chains (see Methods – PUNCH-P). Finally, the newly synthesized proteins were detected by western blotting with streptavidin-HRP and Ponceau S staining for total proteins. We used three procedures: 1) no inhibitors, 2) pretreatment with Actinomycin D 1 h before irradiation and to the duration of the recovery, and 3) pretreatment with Rbin2 for 0.5 h, followed by UV recovery in water at the same concentration for 2 h. The right panel of the western blot was cropped to fit into the image; the original image is shown in **Supplementary Figure S5**. **(B)** The experiment was conducted as in **(A)**, but after purifying the ribosomal fraction using the sucrose cushion, the pellet was dissolved and loaded onto a 10- 50% sucrose gradient. The ribosome analysis was performed as described in the legend of **Figure 1** and under Materials and Methods.

### Translation capacity is induced in *Fusarium* species by UV irradiation at transcription and ribosome maturation levels

Because our experimental approach could not show that UV specifically induces polysomes, we tested directly whether UV increases translation capacity. To this end, we harvested ribosomes from irradiated and non-irradiated cells at different time points after UV recovery and incubated them with biotinylated puromycin. Thus, translation of polypeptide chains can be detected using streptavidin-linked peroxidase (PUNCH-P (Aviner et al., 2013); and Materials and Methods). We observed an increase in the puromycin-peptide signal following irradiation in *F. mangiferae* (**Figure 2A**).

So far, we focused on *F. mangiferae*, but at the transcription level we observed induction of translation-related genes also in *F. oxysporum* (Milo-Cochavi et al., 2019a). Using the same experimental procedures applied to *F. mangiferae*, we detected higher RNA concentrations across the gradient, stronger ribosomal RNA band intensities, and stronger translation capacity following irradiation in *F. oxysporum*. Albeit attenuated in comparison with *F. mangiferae* (**Supplementary Figure S4**).

Ribosome biogenesis comprises several processes, the first of which is rRNA transcription. To test whether UV induces rRNA transcription, we used Actinomycin D to inhibit RNA pol I activity (Perry and Kelley, 1970; Schöfer et al., 1996). 14 hpi-filaments were exposed to 500 nM Actinomycin D one hour before UV irradiation with 200 J/m^2^. After irradiation, the fungi recovered in the presence of the inhibitor for 2 hours. Ribosomes were separated using a sucrose gradient as described above. Actinomycin D completely suppressed UV-induced ribosome biosynthesis; this was observed both at the level of RNA concentration across the gradient and the ribosomal band intensity (**Figure 2B**) (the area under the curve for UV absorbance of the irradiated sample without actinomycin D was 1.18 ± 0.02 higher than with actinomycin D). We also tested the effect of Actinomycin D on UV-induced translation using the PUNCH-P protocol in *F. mangiferae*. We show that UV-induced translation is significantly delayed under Actinomycin D exposure but eventually occurs to a much lesser extent (**Figure 2A**). This observation caused us to suspect that UV also induced ribosome maturation to some extent. To test this, filaments of *F. mangiferae* were treated with 1 µM Rbin2 for 0.5 hour before irradiation (200 J/m^2^) and then incubated with the inhibitor for an additional 2 hours. When we determined translation efficiency using the PUNCH-P approach under these conditions, the UV-induced translation capacity was suppressed with no recovery (**Figure 2A**). The comparison between the dynamics of translation of the cells exposed to Actinomycin D and Rbin2 suggests that UV also induces ribosome maturation, and this is why, even under Actinomycin D exposure, minimal induced translation can be observed.

### Ribosome induction facilitates UV survival and repair of UV-induced DNA damage

Next, we examined whether inducing ribosome biogenesis and translation capacity improves UV survival. To this end, we exposed fungi to 500 nM Actinomycin D and then irradiated them. This time, the cultures were held in water for 24 hours after irradiation with or without Actinomycin D (liquid holding). In *F. mangiferae*, adding Actinomycin D increased the sensitivity of the fungi to 50 J/m^2^ of UV. The effect was observed in all the examined stages of fungal development, but was strongest when 14 hpi filaments were irradiated (**Figure 3A**). Similar results were obtained when *F. mangiferae* cells were irradiated in the presence of 0.4 µg/mL cycloheximide, a protein synthesis inhibitor. Under these conditions, cycloheximide alone did not significantly reduce viability, but when combined with irradiation, viability decreased more than with irradiation alone. This phenomenon was observed in all stages, but the strongest decrease in viability (30%) occurred when 14 hpi filaments were irradiated (**Figure 3B**).

**Figure 3:**
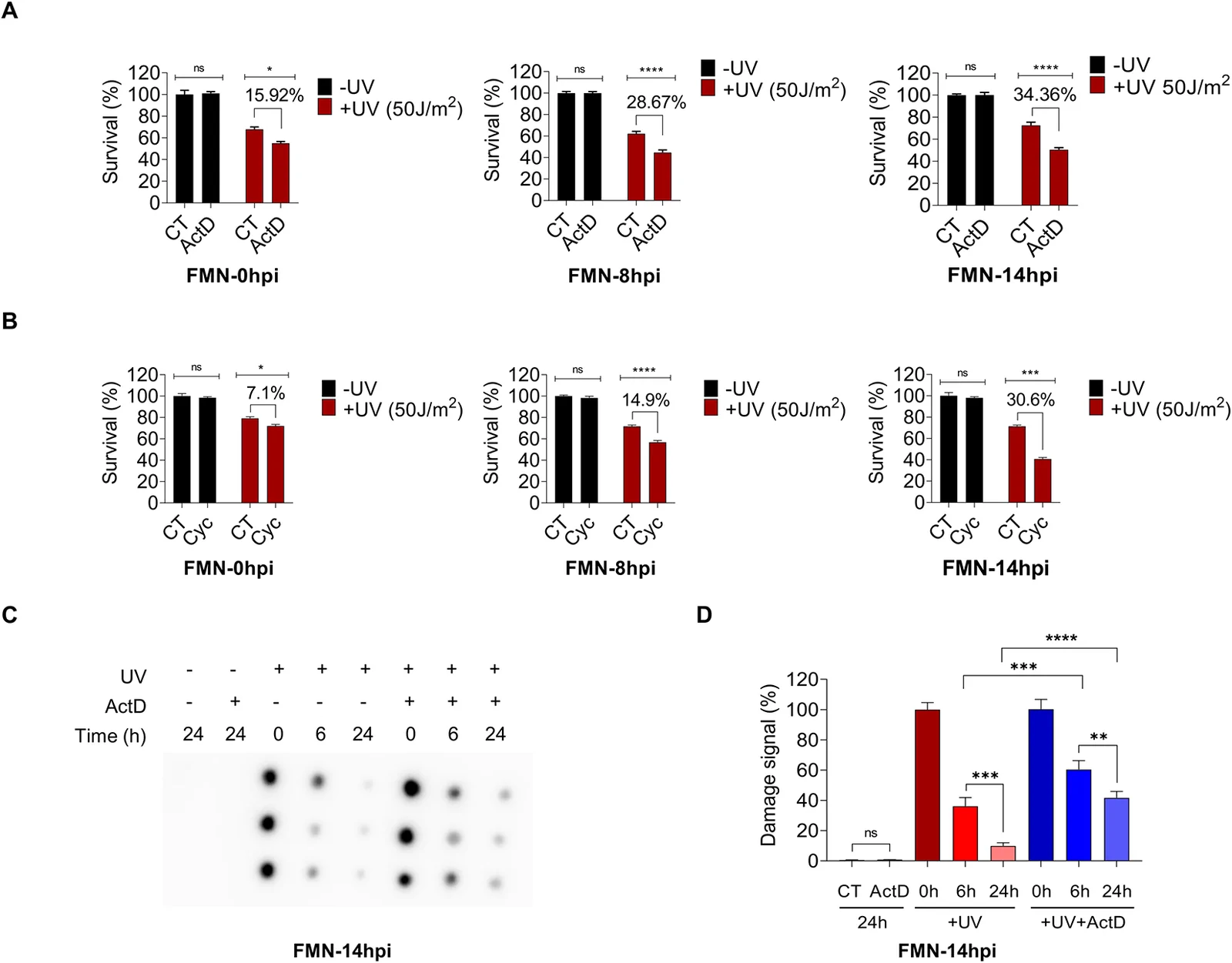
Blocking the UV-induced ribosome biogenesis and translation reduces UV survival and damage repair. *F. mangiferae (*FMN*)* conidia were inoculated for the indicated time points, then irradiated or not. **(A)** One hour before irradiation, the fungi were pretreated with or without Actinomycin D (ActD). After irradiation, the fungi were recovered for 12 h in water with or without Actinomycin D. **(B)** One hour before irradiation, the fungi were pretreated with Cycloheximide (Cyc) or not. After irradiation, the fungi were recovered in water for 6 h, with or without Cycloheximide. For both **(A)** and **(B)**, after UV recovery, the fungi were plated on PDA. Survival was calculated as the ratio of the number of colonies that grew after exposure to Actinomycin D or Cycloheximide to the number of colonies that grew without such exposure (CT). 100% survival refers to the number of colonies without UV or chemical exposure. The results represent the average and SD of three biological replicates. The experiment was repeated three times. Paired-wise, two-way ANOVA was used to calculate significance. *p<0.05, ****p<0.0001. **(C, D)** 14 hpi filaments were irradiated with 100 J/m^2^ and recovered in water with or without Actinomycin D for the indicated time points. After recovery, genomic DNA was isolated; UV lesions were detected by immuno-dot blot assay using antibodies against CPD (see Methods). **(D)** Quantification of damage signal from **(C)**. The damage signal is shown relative to the initial damage inflicted. The damage signal at time point 0 after UV exposure was arbitrarily set to 1. The results are based on an average of six biological replicates, and error bars represent SD. Significance was calculated using Tukey’s multiple comparisons test, **p<0.05, ****p<0.0001.

Next, we wanted to know if UV repair is reduced in irradiated *F. mangiferae* filaments treated with Actinomycin D. In a liquid holding experiment of irradiated filaments for 24 hours, we were able to show that Actinomycin D reduced the repair of pyrimidine dimers. Without Actinomycin D, only 10% of the signal from the UV lesion remained, whereas with Actinomycin D, 40% remained (**Figures 3C, D**). In summary, blocking UV-induced ribosome biogenesis sensitizes *Fusarium* to UV and reduces UV repair. However, the effect, while significant, is relatively modest and reflects a severe shutdown of the DNA damage response, as seen, for example, in the abolition of the SOS response. We note that Actinomycin D may also inhibits mRNA transcription, although less efficiently (Perry and Kelley, 1970; Schöfer et al., 1996). Our working dosage was relatively low and was sublethal. Nevertheless, we cannot exclude the possibility that some of the effect of Actinomycin D on UV repair is due to a direct effect on mRNA transcription. However, even at the mRNA transcription level, the genes induced by UV were related to translation (Milo et al., 2024) (**Supplementary Figure S2**), and the only bona fide UV repair induced following UV is at the translation level (see below).

### mRNAs related to gene expression and proteome health are associated with ribosomes following UV irradiation

Next, we sought to identify the genes whose translation is induced by UV. To this end, we performed an RNA-seq experiment of 14 hpi irradiated filaments two hours after irradiation. We sequenced both the total RNA population and higher- and lower-sucrose gradient fractions (fractions 3–12 and 13-24, respectively) (See Materials and Methods; all data are available in the GEO site under project GSE305365).

We first determined the number of differentially expressed mRNAs by UV at two hours post-irradiation. We compared this number with the number of mRNA induced under the same conditions but at earlier time points post-irradiation (Milo et al., 2024). As expected, the number of different mRNAs induced by UV decreased dramatically over time (**Supplementary Figure S6**). The gene composition also changes: the overlap between induced genes within the first hour post-irradiation was much higher than at 2 hpi (**Supplementary Figure S6**). These findings suggest that, after two hours, the transcriptional response to UV is declining.

We identified mRNAs that are preferentially associated with ribosomes with and without UV irradiation. Supporting our results, UV-irradiated fungi had more than triple the number of distinct mRNA species associated with ribosomes from the bottom of the gradient (430 vs 130). Next, we identified genes differentially associated with ribosomes from the bottom of the gradient only after UV irradiation and analyzed them by function. The genes can be divided into several groups. The largest group comprises genes important for gene expression: transcription, splicing, polyadenylation, ribosome biogenesis, and protein folding (**Figure 4**). In this group, genes such as *ran1* were also found to facilitate the export of ribosomal subunits from the nucleus. This could be the reason why Rbin2 was more effective in suppressing UV-induced translation than Actinomycin D (**Figure 2**). The second group of genes is related to metabolism, which can be further divided into mitochondria/electron chain transfer genes and amino acid metabolism (under the group of tryptophan biogenesis in **Figure 4**, we found genes that are related to the biosynthesis of other amino acids). The third group of genes is related to communication with the environment, either secretion or internalization. Among them, we identified genes related to the endoplasmic reticulum and coated pits. The fourth group seems most relevant to UV exposure, as it includes, among others, DNA repair and cell cycle genes. The automatic classification by the STRING algorithm of TC-NER (transcription-coupled repair) includes, in fact, genes of general NER. The most relevant gene in this group for UV repair is *DDB1 (*Iovine et al., 2011). Another enriched module is double-strand break repair by homologous recombination, including genes such as *rad*55 and *mus*81; DNA synthesis (DNA polymerase delta, psf1, mcm3); and translesion DNA synthesis (DNA polymerase zeta and DNA polymerase kappa). Genes involved in cell cycle checkpoints, such as *cdc6* and the aurora kinase, were also identified (**Figure 4**). The mRNA gene modules associated with lighter ribosomal fractions (3-12) were similar to the heavy ones. They included genes related to translation, mitochondria, and the UV repair gene *DDB1* (**Supplementary Figure S7**). Overall, at two hours post-irradiation, the early induced transcripts appear to be undergoing translation. Indeed, 165 of 430 ribosome-associated genes from the bottom of the gradient were induced by UV within the first hour (**Supplementary Figure S8**). This is not just because these genes are naturally associated with the heavy fraction of the ribosomes; the proportion of these genes associated with un-irradiated heavy ribosomes is significantly lower (**Supplementary Figure S8**).

**Figure 4:**
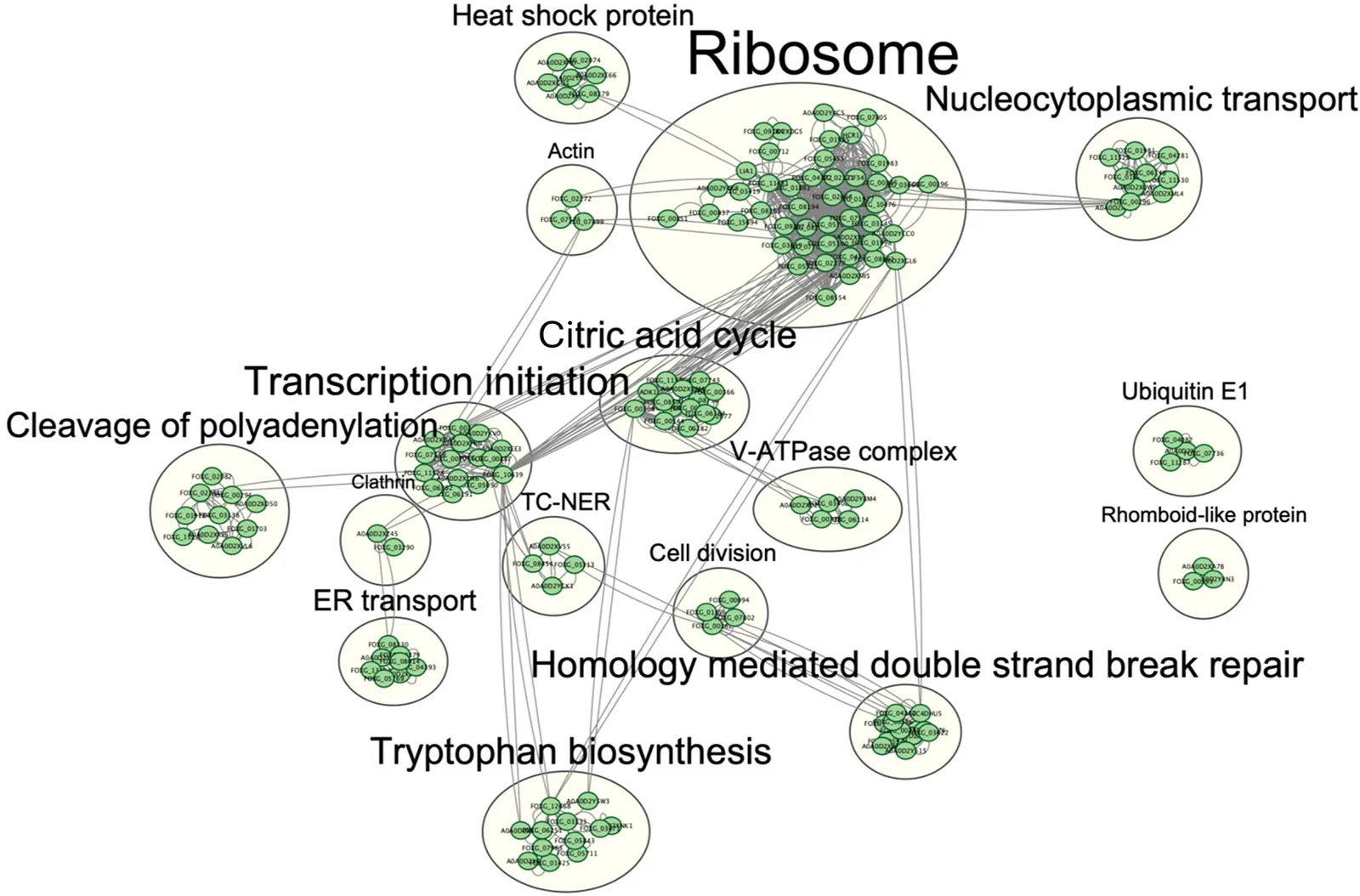
mRNAs associated with ribosomes after UV induction are clustered into gene expression and DNA damage response modules. 14 hpi filaments of *F. mangiferae* were irradiated or not with 200 J/m^2^. Two hours after recovery in water, ribosomes were separated by sucrose density gradient centrifugation as described in **Figure 1** and in the Methods section. RNA was purified from fractions 13-24, as described in the Methods. Next, the mRNA was further purified using poly dT and sent for sequencing. After sequencing, reads that mapped to ribosomal RNA units were removed from the analysis, as described in the Methods. To complete the dataset, total mRNA was purified from the same cultures without further ribosome separation. Genes enriched in heavy ribosome fractions, with and without UV exposure, were identified first (raw data are available at GSE305365). Next, genes from the heavy fraction of the ribosomes that were enriched only after irradiation were identified and analyzed using STRING to reveal UV-regulated translation modules.

### TOR is necessary for full UV-induction of ribosomes

Finally, we sought to understand the mechanism regulating UV-induced translation capacity. The TOR signaling pathway is known to regulate ribosome biogenesis by induction of transcription factors. Therefore, we tested whether inhibiting the TOR signaling pathway with rapamycin would suppress UV-induced ribosome biogenesis and translation-related genes. To this end, we pretreated *F. mangiferae* filaments with 5 µg/mL rapamycin two hours before irradiation. We harvested the RNA 30 minutes post-irradiation and sequenced it. As a control, we harvested RNA from untreated filaments and from filaments treated with rapamycin but not irradiated.

We first tested how rapamycin affects UV-induced gene expression. We compared UV-induced genes under rapamycin exposure (UV+ rapamycin) with no treatment, as we previously determined (Milo et al., 2024). Pretreatment with rapamycin completely changes UV’s effect on gene expression. There was very little overlap between the genes that were induced by UV with rapamycin and those that were induced without it (**Supplementary Figure S9A**). Importantly, while UV induces ribosome biogenesis genes in the absence of rapamycin [**Supplementary Figure S2** (Milo et al., 2024)], we did not observe this effect when cells were exposed to rapamycin. However, UV induces gene expression even when filaments are pretreated with rapamycin. After removing the contribution of rapamycin to gene induction, about 1000 genes are induced by UV compared with untreated filaments (no UV, no rapamycin). Functional characterization of UV-induced genes during rapamycin exposure reveals carbon and amino acid metabolic modules (Fatty acid metabolism, pyruvate and ethanol metabolism) (**Supplementary Figure S9B**). Next, to directly test the effect of UV under rapamycin exposure conditions, we compared gene expression under rapamycin exposure, with or without UV. Interestingly, here we do observe induction of ribosome biogenesis and translation-related genes (**Supplementary Figure S10**). We also observed induction of genes related to nitrogen and amino acid metabolism. Several modules are downregulated by UV, including splicing and mitosis, but what we find most interesting are genes related to gluconeogenesis (**Supplementary Figure S11**). To sum up, it seems that, with rapamycin pre-treatment, UV alters filament metabolism.

Next, we performed a UV survival experiment as we did with Actinomycin D and Cycloheximide. In *F. mangiferae*, rapamycin did not reduce UV survival at the filament stage (**Figure 5C**, comparison of the effect of UV between 0 and 0.5 µg/mL rapamycin). Intrigued by these results, we measured rapamycin toxicity with and without UV irradiation. Surprisingly, UV exposure rescued rapamycin toxicity across all developmental stages, especially at the germling (8 hpi) stage (**Figures 5A–C**).

**Figure 5:**
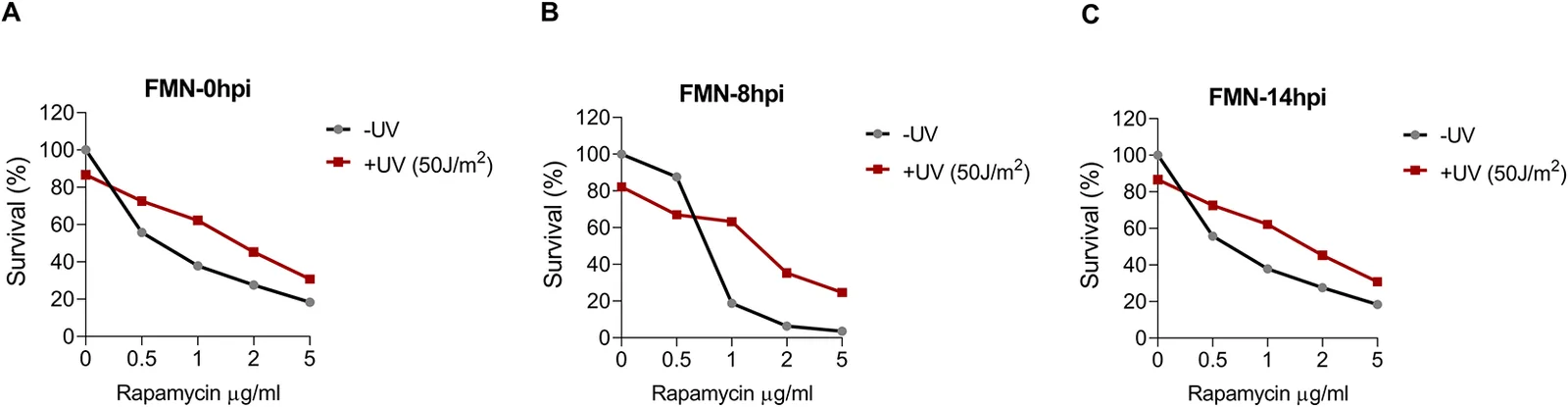
UV rescues *F. mangiferae* from rapamycin toxicity. *F. mangiferae* were inoculated for 0 **(A)**, 8 **(B)** or 14 **(C)** hours in PDB. Then the fungi incubated were in water for 1 h with the indicated doses of Rapamycin. Next, the cultures were either irradiated or not and recovered in water containing the indicated doses of Rapamycin for 12 h. Cells were spread over PDA plates. Survival was calculated by dividing the number of colonies that grew after each treatment by the number of colonies in the culture that was not irradiated or treated with Rapamycin (considered as 100%). The results represent the average and standard deviation (SD) of 3 biological replicates.

## Discussion

### Why UV-induced translation and why in fungi?

Here, we show that UV induces translation in two *Fusarium* species (**Figures 1**, **2**; **Supplementary Figure S4**). This contrasts with many reports on the negative effects of DNA damage and other stresses on ribosome biogenesis. The question is why? Unlike the SOS response, our results do not support the induction of many UV-DNA repair genes (Maslowska et al., 2019) (**Figure 4**; **Supplementary Figure S7**), although *DDB1* is induced at the translational level. We observed a positive and significant effect of ribosome biogenesis on UV survival and DNA repair, but it is not comparable to the effect of the SOS pathway in bacteria [**Figure 3**; (Keller et al., 2001)]. Several post-replication DNA repair genes are specifically associated with ribosomes following UV (**Figure 4**; **Supplementary Figure S7**). However, mRNAs related to transcription, translation, protein secretion, and other functions are also significantly more associated with ribosomes following UV (**Figure 4**; **Supplementary Figure S7**). We think the UV-induced increase in translation capacity compensates for the negative effect of UV on transcription and, consequently, translation (Nieto Moreno et al., 2023). As a support mechanism, the association of mRNA encoding housekeeping proteins, including chaperones, with ribosomes is enriched following UV exposure to maintain homeostasis. We are currently testing whether other ways to block mRNA transcription elicit the same response, or whether the DNA damage response strictly mediates it. An alternative model of UV-induced translation suggests the signal is not DNA damage and stalled transcription, but RNA damage and stalled translation (Wu et al., 2020). According to this model, destruction of damaged mRNA and rRNA requires *de novo* transcription. In light of this model, Actinomycin D affects UV repair or survival through both rRNA and mRNA transcription. In our view, the best way to differentiate between the models is to selectively disrupt transcription-coupled repair or ribosome/RNA quality control (Xrn1, Hel2) and determine where the effect on UV-induced translation is greater.

Why was UV induction of ribosomes never observed in other organisms? We suggest this relates to the (in)ability of *Fusarium* species to overcome UV effects on transcription. In many organisms, from *E. coli* to humans, transcription-coupled repair is dedicated to overcoming DNA damage-induced stalling of transcription (Gaul and Svejstrup, 2021). We hypothesize that transcription-coupled repair in *Fusarium* species is less efficient than in other organisms and, consequently, that transcription resumption following DNA damage is slower. To support this, we previously reported that DNA damage induces mRNA levels of components of the basic mRNA transcription machinery (Milo-Cochavi et al., 2019b). Here we observed that the association of these mRNAs with ribosomes is increased following UV exposure (**Figure 4**). This could be the response in *Fusarium* to the destruction of the stalled transcription apparatus in case transcription-coupled repair is inefficient, or it could be that normal transcription machinery is not enough to trigger TCR and more transcription initiation is needed. It was previously demonstrated that waves of RNA polymerase II are released from pause to transcription elongation following UV exposure, in what appears to be a mechanism to reduce mutagenesis (Lavigne et al., 2017). Induction of the transcription machinery can also support a model of compensation for RNA damage removal.

Another explanation for why we observed UV-induced ribosome biogenesis in *Fusarium* but not in other species may be the developmental biology of filamentous fungi. UV-induced ribosome biogenesis is observed at 14 hpi, when filaments are forming (**Figures 1**, **2**; **Supplementary Figure S4**). While growth at 14 hpi is still fast, most nuclei in the filament are already dormant. Although most nuclei should be mitotically dormant, among the genes associated with the heavy fractions of the ribosomes following UV are those related to the cell cycle, specifically mitosis. This may suggest that different nuclei translate distinct sets of genes following UV exposure, a heterogeneity absent in *E. coli* or yeast cells. One aspect to consider is the division of labor and the interplay between TOR signaling and PKA in ribosome biogenesis (Guerra et al., 2022; Plank, 2022). The balance between these two pathways may change during fungal development in ways not observed in organisms studied to date. *Fusarium mangiferae* is more sensitive to rapamycin at the germling stage than the filament stage (**Figure 5**). It is possible that when TOR signaling is dominant, UV induction is insignificant, but when the balance is changed towards PKA, either by TOR inhibition or during development, UV induction is more significant. UV-induced PKA signaling may activate pathways that, in turn, activate TOR, which is ultimately required for UV-induced ribosome biogenesis (**Supplementary Figure S10**).

### What could be the possible mechanism of UV-induced translation?

In addition to the questions raised above regarding the trigger for UV-induced translation, we also do not know how the signal is transduced from damaged nucleic acids to PKA/TOR signaling (or else).

Translation requires a lot of energy. Consistent with this, mitochondrial genes are induced at the transcriptional and ribosome-association levels following UV exposure [**Supplementary Figure S2**; **Figure 4**; **Supplementary Figure S7** (Milo-Cochavi et al., 2019a; Milo et al., 2024)]. What metabolic adjustments do the fungi need to make to enable this energy production? When TOR is repressed by rapamycin, many of the differentially expressed genes are involved in metabolism (**Supplementary Figures S10**, **11**; **Supplementary Tables S2**–**S4**). Could UV induce metabolic rewiring that increases the rate of energy production? And if so, how?

### How general is the UV-induced translation?

Is UV the only stress to induce ribosome biogenesis in *Fusarium* species? Our favorable model is that stalled transcription bubbles trigger UV-induced translation. Our results with methyl methanesulfonate were inconclusive and were not included here. Therefore, we only expect bulky lesions to induce translation. Yet, this hypothesis should be tested with more DNA-damaging agents and additional stressors. Reactive oxygen species (ROS), are important stressors and signaling molecules in fungi (Zhang et al., 2020; James et al., 2024). While they inhibit translation in yeast (Meydan et al., 2023), their effects in *Fusarium* species remain unknown. In addition, it remains to be determined whether UV-induced translation is shared among other *Fusarium* species or other filamentous fungi.

### How does the fungal physiology affect UV-induced translation?

At the transcriptional level, UV induction of ribosome biogenesis and translation-related genes is immediate and occurs within minutes of growth in rich media (Milo-Cochavi et al., 2019a; Milo et al., 2024). However, we show that the response continues even after two hours of recovery in water (**Figures 2**, **3**, **5**). How adding nutrients during recovery affects the response is unknown. Back to the hypothesis that UV boosts translation by rewiring metabolism and activating TOR signaling; will it differ if recovery occurs in rich media?

### Could UV-induced translation be exploited to treat fungal diseases?

Finally, from a plant pathology standpoint, the commitment to ribosome biogenesis is very high and requires a lot of energy. This energy consumption may increase fungi’s vulnerability to stress. Can this be used to fight *Fusarium* pathogens?

## Data Availability

The original contributions presented in the study are publicly available. This data can be found at the NCBI with accession numbers: GSE305365 and GSE254119.

## Supplementary material

Supplementary Figure S1 RNA pattern before and after separation on 35% sucrose cushion. Total RNA isolated from fungal extract before loading on the sucrose cushion (T) and RNA that was recovered from the pellet after ultracentrifugation (P) were separated on a TAE agarose gel. Size markers in base pairs.

Supplementary Figure S2 Modules related to translation are induced by UV in *F. mangiferae* when filaments but not germlings are irradiated. Conidia of *F. mangiferae* were inoculated for 8 or 14 h in PDB medium. The germlings or filaments were harvested, re-suspended in water, and irradiated with 200 J/m^2^ after recovery for 0, 30, or 60 minutes in complete darkness. We purified and sequenced the mRNA; raw data are available in GSE254119. The UV-differentially expressed genes across all time points were pooled and analyzed using STRING to identify up- and down-regulated modules.

Supplementary Figure S3 UV causes nuclear division delay in filaments of *F. mangiferae.* **(A)**. Representative images of *F. mangiferae* (FMN), 14 hpi filaments were irradiated with 200 J/m^2^ UV and then were released for two to six hours in PDB. Fluorescence indicating nuclear fragmentation by DAPI staining. After UV exposure, fungal cells were fixed with 4% paraformaldehyde in phosphate-buffered saline (PBS) for one hour at room temperature. Then, the filaments were washed before dehydrated in 75% ethanol overnight. Samples were then washed twice with PBS before DAPI staining (1 μg/ml). Scale bar, 100µm. **(B)** Distribution of nucleus numbers in filaments of *F. mangiferae*. Significance was calculated using one-way ANOVA, Tukey test, *p<0.05, ****p<0.0001

Supplementary Figure S4 UV induces ribosome biogenesis and translation capacity in filaments of *F. oxysporum.* Ribosome gradient profiles of *F. oxysporum* (FOL)14 hpi **(A)** irradiated with 50 and 200J/m^2^ UV doses. The RNAs from each fraction of ribosome gradient were separated on 2% agarose gels (top panel); ribosome intensity was calculated based on the strongest band signal in the control sample (signal intensity as 1) (similarly to what is described in the legend of **Figure 1** and in Materials and Methods). A254 absorbance value across the gradient (low panel). **(B)** The effect of UV on translation capacity. Ribosome of *F. oxysporum* were isolated after UV exposure of 14 hpi filaments. Next, the ribosomes were labeled by Biotin-Puromycin for the indicated times and under the indicated conditions and detected by western blotting using streptavidin-HRP (similarly to what is described in the legend of **Figure 2A**).

Supplementary Figure S5 Rbin2 suppresses UV-induced translational capacity in *F. mangiferae* (FMN). This is the full image of the cropped image presented in **Figure 2**.

Supplementary Figure S6 Delayed response of *F. mangiferae* filaments to UV: the early response differs in the number of induced genes and in gene composition. The number of genes induced by UV after irradiation of filaments. The numbers for 0, 30, 60 and 120 minutes after irradiation recovery times are shown. The data for the early time points (0-60) are taken from (Milo et al., 2024) (see also the legend to **Supplementary Figure S2**). The data for the later time point (120 min) were collected as described in Materials and Methods and in the legend to **Figure 4**. The overlap between the gene lists was created by Venny 2.1.

Supplementary Figure S7 mRNA associated with ribosomes after UV induction are clustered into gene expression and DNA damage response modules. 14 hpi filaments of *F. mangiferae* were irradiated or not with 200 J/m^2^. Two hours after recovery in water, ribosomes were separated using a sucrose gradient as described in **Figure 1** and under Methods. RNA was purified from fractions 3–12 as described in Methods. Next, mRNA was further purified using poly dT and sent to sequencing. After sequencing, reads mapped to ribosomal RNA units were removed from the analysis as described in Methods. To complete the dataset, total mRNA was purified from the same cultures without further ribosome separation. We first identified genes enriched in fractions 3-12, with and without UV. Next, we identified genes that are enriched only in irradiated fractions 3-12. We analyzed these genes using STRING to identify modules likely induced by UV at the translational level.

Supplementary Figure S8 Early UV-induced mRNA species are more associated with heavy ribosome fractions of UV-irradiated filaments than non-irradiated filaments. Comparison 1 presents a Venn diagram (Venny 2.1) of free mRNA induced by UV between 0–60 minutes of recovery time and mRNAs associated with fractions 13-24 (heavy) 120 minutes after irradiation. Comparison 2 presents a Venn diagram of the free mRNAs that were induced by UV between 0–60 minutes of recovery time and mRNAs that are associated with fractions 13-24 (heavy) 120 minutes of recovery with no irradiation (Ct). UV-induced genes tend to be more associated with heavy fractions after UV; Fisher’s exact test: 0.0023. For early time points, total free mRNA were analyzed as described in the legend of **Supplementary Figure S2**. For RNA from fractions 3–12 of the gradient, the experiment was done as described in the legend of **Figure 4**.

Supplementary Figure S9 UV induction of ribosome biogenesis genes is severely affected by rapamycin. **(A)**. Venn diagram of *F. mangiferae* UV-induced genes with and without 5 μg/mL rapamycin. Conidia were inoculated into PDB for 13 h, treated with rapamycin for 1 h, irradiated with 200 J/m^2^, and recovered for 30 minutes in water with rapamycin. RNA was purified from treated and untreated filaments. We compared genes induced under these conditions with those induced by UV in the absence of rapamycin, as described in Milo et al.; Fungal Biology, 2024. **(B)**. The genes up-regulated in the comparison of UV+rapamycin versus untreated were compared with those induced by rapamycin alone using Venn. *F. oxysporum* orthologs of the UV-specific induced genes were analyzed using STRING. Functional clusters were presented using Cytoscape.

Supplementary Figure S10 UV-induced genes under rapamycin exposure show a modest induction of translation-related genes. *F. oxysporum* orthologs of the *F. mangiferae* UV-induced genes by UV after pretreatment with rapamycin. Genes were clustered into functional groups using STRING. Functional clusters were presented using Cytoscape. The experiment was performed as described in the legend of **Supplementary Figure S9**.

Supplementary Figure S11 UV-suppressed genes under rapamycin exposure show a reduction in transcription-related genes. *F. oxysporum* orthologs of the *F. mangiferae* UV-suppressed genes after pretreatment with rapamycin. Genes were clustered into functional groups using STRING. Functional clusters were presented using Cytoscape. The experiment was performed as described in the legend of **Supplementary Figure S9**.
